# Diabetes Mellitus is Associated With Low Secretion Rates of Immunoglobulin A in Saliva

**DOI:** 10.2188/jea.JE20140088

**Published:** 2015-07-05

**Authors:** Junko Oikawa, Shigekazu Ukawa, Hideki Ohira, Takashi Kawamura, Kenji Wakai, Masahiko Ando, Akira Hata, Akiko Tamakoshi

**Affiliations:** 1 Department of Public Health, Hokkaido University Graduate School of Medicine, Sapporo, Japan; 2 Department of Public Health, Chiba University Graduate School of Medicine, Chiba, Japan; 3 Department of Psychology, Graduate School of Environmental Studies, Nagoya University, Nagoya, Japan; 4 Kyoto University Health Service, Kyoto, Japan; 5 Department of Preventive Medicine, Nagoya University Graduate School of Medicine, Nagoya, Japan; 6 Center for Advanced Medicine and Clinical Research, Hospital, Nagoya University, Nagoya, Japan

**Keywords:** diabetes mellitus, secretory immunoglobulin A, saliva

## Abstract

**Background:**

The association between diabetes mellitus (DM) and low secretory immunoglobulin A (s-IgA) secretion rates is one mechanism suspected of influencing susceptibility to infections among DM patients. However, several studies have shown contradictory results. We examined these two factors to seek evidence of an association among older people.

**Methods:**

We analyzed a prospective cohort of 2306 subjects (1209 men and 1097 women) around 64 years old from the New Integrated Suburban Seniority Investigation (NISSIN) Project in Nisshin, Japan. DM statuses were ascertained from levels of fasting plasma glucose and HbA_1c_, and s-IgA secretion rates were obtained from 5-min saliva samples. We used an analysis of covariance adjusted for possible confounders to compare s-IgA secretion rates according to DM status.

**Results:**

s-IgA secretion rates in DM participants were lower than in those classified as normal (18.6 µg/min vs 15.0 µg/min, *P* = 0.03), even after elimination of the effects of possible confounders.

**Conclusions:**

DM was associated with lower s-IgA secretion rates. This suggests that lower s-IgA levels may be a mechanism of susceptibility to infection in individuals with DM.

## INTRODUCTION

Secretory immunoglobulin A (s-IgA), the predominant immunoglobulin in secretions of the mucosal immune system, plays an important primary role in antigen excretion.^1^ It is found in saliva, intestinal secretions, bronchoalveolar lavage fluid, urine, tears, and other mucosal fluids. It inhibits attachment and replication of pathogenic microorganisms, thus preventing colonization of these pathogens. It is also capable of neutralizing toxins and viruses.^1^^,^^2^ A study of humans with mucosal humoral immunodeficiencies suggests that the absence of s-IgA leads to an increase in mucosal infections and respiratory tract infections.^3^ Moreover, suppression of s-IgA is associated with increased incidence of upper respiratory tract infection (URTI) in elite athletes^4^^,^^5^ and healthy adults.^6^

Diabetes mellitus (DM) is associated with an increased risk of morbidity from infectious diseases, such as pneumonia and urinary tract infections.^7^^,^^8^ Several previous studies have reported that DM patients have impaired chemotaxis of the immune cells, defective phagocytosis of macrophages, and increased production of free radicals.^9^^,^^10^ Hyperglycemia can cause microangiopathy, which can lead to ulceration, secondary infection,^11^ and neuropathy. Microangiopathy may also result in impaired bladder emptying, which increases susceptibility to urinary tract infections.^12^ Several mechanisms seem to cause susceptibility to infections in patients with diabetes. A low s-IgA secretion rate is suspected to be one of these mechanisms; however, previous studies have shown contradictory results.^13^^,^^14^ Branco-de-Almeida et al reported that DM patients had lower mean levels of s-IgA than non-DM patients,^13^ while Yavuzyilmaz et al reported that DM patients had higher mean levels of s-IgA than healthy controls.^14^ These studies^13^^,^^14^ used small and limited community samples and failed to control for factors such as age, educational background, smoking, physical activity, or periodontitis, which are now known to be related to both s-IgA secretion and DM.^15^^–^^17^

We investigated the association between DM and s-IgA secretion rates using data from the New Integrated Suburban Seniority Investigation (NISSIN) Project, a large community sample of older people.

## MATERIALS AND METHODS

### Study population

This cross-sectional study was conducted using baseline data from the NISSIN Project, an age-specific prospective cohort study in Nisshin City, which is located near Nagoya City, Japan. Using the basic resident registry of Nisshin City, residents aged 64 years were invited to attend a free health check-up every April from 1996 through 2005; ultimately, 3073 subjects were recruited into this cohort (43.9% of the eligible population). Details of the NISSIN Project have been described elsewhere.^18^ Subjects were given a comprehensive medical and dental check-up in the early morning after an overnight fast. Participants in the present study were limited to those recruited from 1998 until 2005, as saliva samples were collected during this period.

Of the 2579 subjects that we approached, 2557 agreed to participate. From these, 2492 saliva samples (from 1275 men and 1217 women) were obtained. We excluded 183 participants because too little saliva was collected for assay. A further three participants were excluded due to missing fasting plasma glucose (FPG) and glycated hemoglobin (HbA_1c_) data. Thus, a total of 2306 subjects (1209 men and 1097 women) were analyzed.

Until 2001, oral consent was obtained using an opt-out approach, and written consent by an opt-in approach was used thereafter. We followed the checklist of the STROBE (STrengthening the Reporting of OBservational studies in Epidemiology) guidelines.^19^ This study was approved by the Ethics Committees of Nagoya University Graduate School of Medicine, the National Center for Geriatrics and Gerontology of Japan, the Aichi Medical University School of Medicine, and the Hokkaido University Graduate School of Medicine.

### Sample collection

The medical check-up consisted of clinical and laboratory blood testing, including measurement of FPG and HbA_1c_. All clinical tests were performed in a single laboratory. A self-administered questionnaire was used to assess demographic and lifestyle factors. Demographic factors included gender and educational background. Lifestyle factors included smoking status and physical activity.

Dentists performed a dental check-up, and community periodontal index (CPI) codes were recorded as an estimate of periodontitis. CPI codes of 0–4 were assigned: 0 for healthy cases; 1 for bleeding on probing; 2 for presence of calculus; 3 for a probing pocket depth of 4–5 mm, and 4 for a probing pocket depth of ≥6 mm. The highest CPI code recorded for any of each participant’s teeth was applied to that participant.

### Definition of diabetes mellitus status

Levels of HbA_1c_ were determined via the Japan Diabetes Society (JDS) method. JDS HbA_1c_ (%) was converted to the internationally used HbA_1c_ (%) measurement as defined by the National Glycohemoglobin Standardization Program (NGSP), using the following formula: NGSP (%) = 1.02 × JDS (%) + 0.25 (%). DM was then classified as:

Normal: FPG <6.1 mmol/l and HbA_1c_ (NGSP) <5.6% (38 mmol/mol)^20^
Pre-DM: 6.1 mmol/l ≤ FPG <7.0 mmol/l; or 5.6% (38 mmol/mol) ≤ HbA_1c_ (NGSP) <6.5% (48 mmol/mol)^20^
DM: FPG ≥7.0 mmol/l or HbA_1c_ (NGSP) ≥6.5% (48 mmol/mol),^20^ or use of medication for DM

Use of medication for DM was obtained via the question: “Are you taking any oral medications for DM?”. We did not include a question about subcutaneous injection of insulin.

### Salivary s-IgA analysis

Salivary s-IgA analysis was performed using the same method as previously reported.^21^ Briefly, to determine the volume and concentration of saliva, samples of unstimulated saliva were collected with two cotton swabs (Salivettes; Sarstedt Ltd., Leicester, UK) prior to the dental check-up. The swabs were placed under the tongue of each participant and removed after 5 minutes, and the saliva was extracted from the cotton by centrifugation. Then we stored the saliva samples in tubes at −20°C until analysis. An enzyme-linked immunoabsorbent assay (ELISA) was performed to measure the s-IgA concentration in the saliva using an IgA test (MBL Inc., Santa Clara, CA, USA). ELISA was performed once a year from 1998 until 2005, and the laboratory personnel conducting the tests did not have knowledge of the diabetes status of any samples.

### Statistical method

The s-IgA secretion rates (µg/min) were calculated as the product of saliva volume (mL/min) and s-IgA concentration (µg/mL). Because data did not fall into a normal distribution, the s-IgA secretion rate was subjected to log transformation before analysis. This strategy has been adopted in other studies.^22^^,^^23^

To compare s-IgA secretion rates according to DM status, we used an analysis of covariance and adjusted for variables such as gender, assay batch (attended year), educational background (under junior high school, high school, college or higher, and unknown), smoking status (current smoker, ex-smoker, never smoker, and unknown), physical activity (almost none, more than once a week, and unknown), periodontitis (yes: CPI = 4; no: CPI < 4), and intake of any medication (yes or no). We considered these variables as confounding factors based on previous reports.^15^^–^^17^^,^^22^^–^^25^ Since the assay batch variable reflected inter-assay variation, it could have been a potential confounder. Tests for linear trends were conducted to assess associations between the original continuous variables of HbA_1c_ and s-IgA secretion rate. All *P* values were two-tailed and considered statistically significant when *P* < 0.05. All statistical analyses were performed using JMP Pro 10.0.2 software (SAS Institute Inc., Cary, NC, USA).

## RESULTS

Of 1209 men and 1097 women, 176 men (14.6%) and 77 women (7.0%) were categorized as having DM. Characteristics of those with DM did not differ between subjects who were eligible for participation and who were excluded for producing too little saliva to assay (data not shown). Table shows the distribution of participants according to DM status. The proportion of patients with DM was low among those with relatively high educational background. Never having smoked tended to be associated with normal DM status. Physical activity and CPI had no association with DM status in this study. Medication intake was positively associated with DM status.

**Table.** Distribution of participants according to DM status^a^ (*n* = 2306)

	Normal (*n* = 1262)	Pre-DM (*n* = 791)	DM (*n* = 253)
Gender			
Male	635 (50.3)	398 (50.3)	176 (69.6)
Female	627 (49.7)	393 (49.7)	77 (30.4)
Educational background			
Junior high school or lower	395 (31.3)	235 (29.7)	75 (29.6)
High school	537 (42.5)	368 (46.5)	110 (43.5)
College or higher	316 (25.0)	184 (23.3)	65 (25.7)
Smoking status			
Never smoker	712 (56.4)	425 (53.7)	101 (39.9)
Ex-smoker	336 (26.6)	222 (28.1)	104 (41.1)
Current smoker	214 (17.0)	143 (18.1)	48 (19.0)
Physical activity			
Almost none	612 (48.5)	393 (49.7)	109 (43.1)
More than once a week	648 (51.4)	397 (50.2)	144 (56.9)
Community periodontal index			
<4	1181, 93.58	742 (93.8)	238 (94.1)
4	81, 6.42	49 (6.2)	15 (5.9)
Receiving medication			
No	540 (42.8)	314 (39.7)	61 (24.1)
Yes	722 (57.2)	477 (60.3)	192 (75.9)

DM, diabetes mellitus; FPG, fasting plasma glucose.

Values are expressed as numbers (%). Some factors have missing values, so the totals are not always 100%.

^a^DM status was classified as, Normal: FPG < 6.1 mmol/l and HbA1c < 5.6% (38 mmol/mol); pre-DM: 6.1 mmol/l ≤ FPG < 7.0 mmol/l, or 5.6% (38 mmol/mol) ≤ HbA1c < 6.5% (48 mmol/mol); DM: FPG ≥ 7.0 mmol/l or HbA1c ≥ 6.5% (48 mmol/mol).

The median s-IgA secretion rate was 35.7 µg/min (min: 0.0310 µg/min, max: 568 µg/min). Mean saliva volume did not differ significantly between normal, pre-DM, and DM participants (data not shown).

**Figure 1.**
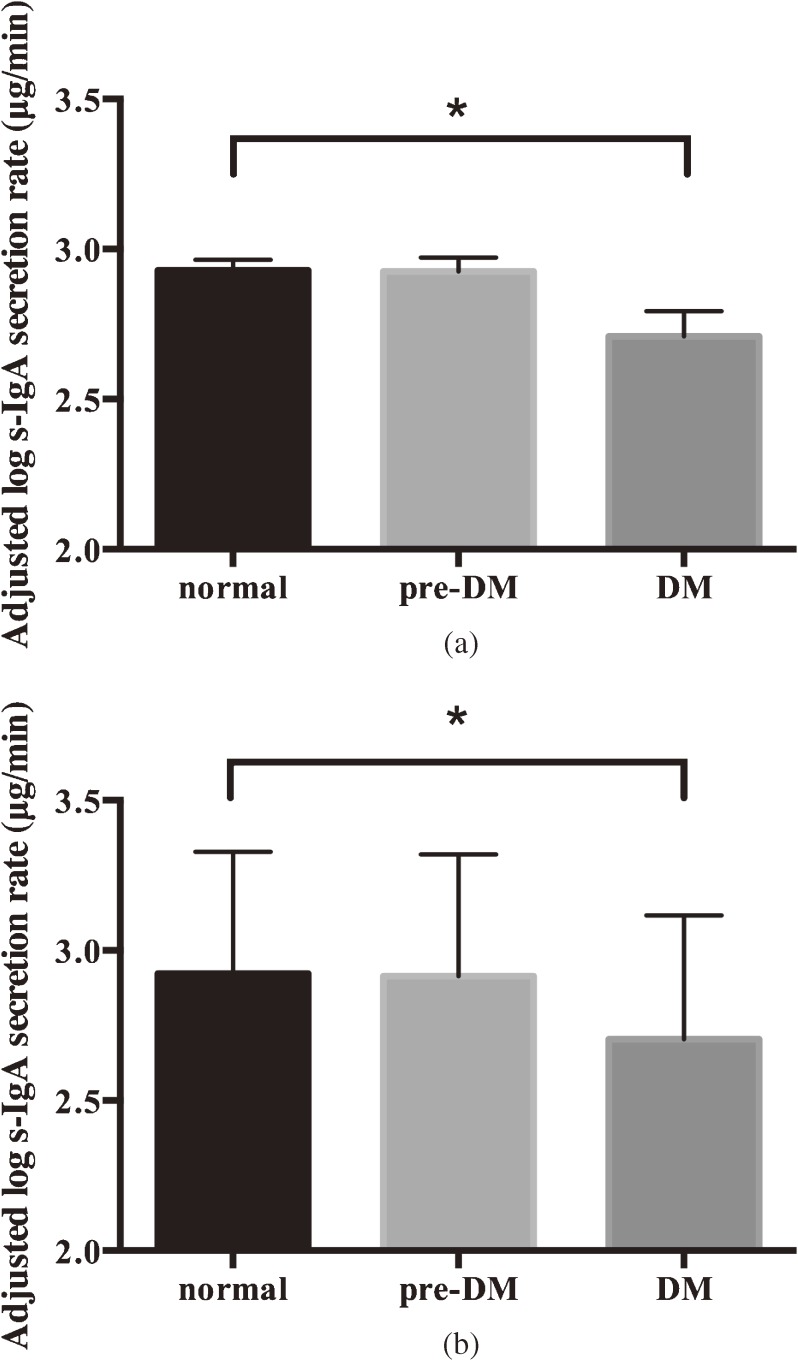
Mean (SD) log s-IgA secretion rate according to diabetes mellitus (DM) status. Analyses shown in Figure 1a were controlled for gender and assay batch. Analyses in Figure 1b were controlled for gender, assay batch, educational background, smoking status, community periodontal index, medication, and physical activity. *P < 0.05.

Figure 1a shows the mean log s-IgA secretion rates according to DM status after adjusting for minor confounders, such as gender and assay batch. s-IgA secretion rates were lower among participants with DM than those classified as normal (18.6 µg/min vs 15.0 µg/min, *P* = 0.03), even after elimination of the effects of all possible confounders, such as assay batch, gender, educational background, smoking status, physical activity, CPI, and medication intake (Figure 1b). Low s-IgA secretion rate also had a linear relationship (*P* for trend = 0.03) with high HbA_1c_.

## DISCUSSION

The present study is the first to show an association between DM and low s-IgA secretion rates in a large sample of older people. The findings are important because of the increasing number of DM patients and deaths from pneumonia in older Japanese people.^26^^,^^27^

This cross-sectional study was developed from a previous case-control study that showed that DM patients presented with lower s-IgA levels than non-DM patients.^13^ Moreover, our study assessed a large community sample of old people of almost the same age controlled for possible confounders. Potential mechanisms involved in the association between DM and low s-IgA secretion rate are not clear. However, a recent study revealed a link between low levels of peritoneal B-1a cells and low production of IgM in DM mice.^28^ Experiments in vitro have confirmed the causal relationship of high concentrations of glucose on reduced secretion of total IgM.^28^ Similar mechanisms may work to induce lower s-IgA secretion rates in human DM patients’ salivary glands. More female participants (*n* = 121) were excluded because too little saliva was collected for assay than male participants (*n* = 62). It is possible that more women had low salivary flow and suffered from xerostomia than men.^25^^,^^29^

Although the differences in s-IgA secretion rates between normal status participants and those with DM status were small, they were consistent and remained significant following adjustment for several variables known to have some influence on the s-IgA secretion rate. They are also of the same order of magnitude reported previously for s-IgA secretion rates and stress.^22^^,^^23^ Nevertheless, whether associations of this magnitude are of clinical significance remains unclear. A previous cohort study, although based on a younger and smaller sample of athletes than the present study, has shown that subjects prone to developing URTI had significantly lower saliva s-IgA secretion rates than URTI-free subjects.^5^ This suggests that our findings might have some clinical importance. Further prospective studies are needed to clarify the nature of the associations between s-IgA secretion rates and the development of other infectious diseases such as URTI, pneumonia, and urinary tract infections.

The present study has several limitations. First, it is cross-sectional and thus could not show a causal relationship between hyperglycemia and low s-IgA secretion rates. Second, we did not obtain information about the varieties or frequencies of medications taken by participants. Many drugs, such as diuretics, antihistamines, and anti-hypertension drugs, are known to reduce saliva volume as an adverse side effect.^23^^,^^30^ Subjects with DM status might take more medications, which may itself result in reduced saliva volume. However, in a restricted analysis of participants who took no medication, DM was still associated with low s-IgA secretion rate (data not shown). Therefore, the lack of detailed information on medications taken might not have affected our results. Third, the proportion of participants strongly suspected to be DM patients (HbA1c ≥6.1% or receiving medication for DM) in our study was 13.7% (17.0% of men and 10.1% of women), which is close to the national estimate in a similar age group in 2006 (13.6%; 14.7% in men and 12.8% in women).^31^ However, the proportion of participants who might be DM patients (HbA1c 5.6%–6.1%, without receiving medication for DM) was 21.2% (20.7% in men and 21.9% in women) in our study, which is slightly higher than the national estimate (15.4%; 14.4% in men and 16.1% in women). Of note, however: differences in the prevalence of diabetes or pre-diabetes would not affect the association between DM and s-IgA secretion rate.

In conclusion, DM was associated with low s-IgA secretion rate in the present study. Given the prominent role of s-IgA in immune defense on mucosal surfaces and the frequency with which infections are initiated on these surfaces, s-IgA affords a pathway by which higher plasma glucose levels might increase susceptibility to infectious diseases in DM individuals. This result suggests that low s-IgA levels may be a mechanism explaining susceptibility to infection in DM individuals.
